# Outside the box: comparative stem cell insights for fertility biotechnology and conservation

**DOI:** 10.1016/j.xfre.2025.02.002

**Published:** 2025-04-15

**Authors:** Ashlee M. Hutchinson, Antonia Weberling, Yoshinori Endo, Rhishikesh Bargaje

**Affiliations:** a Revive & Restore, Sausalito, California; b Centre For Reproductive Health, Hudson Institute of Medical Research, Clayton, Victoria, Australia; c All Souls College, University of Oxford, Oxford, United Kingdom; d Department of Obstetrics, Gynecology & Reproductive Sciences, University of California San Diego, La Jolla, California; e San Diego Zoo Wildlife Alliance, Escondido, California; f Conception Bioscience, Berkeley, California

**Keywords:** Stem cell, conservation, in vitro gametogenesis, comparative biology, biodiversity

## Abstract

Leveraging comparative biology could lead to breakthrough discoveries in the development of fertility technologies while supporting conservation efforts. Here, we discuss examples and opportunities to learn from stem cell systems in nontraditional animal models, primarily from the amniote phylum, focusing on challenges and insights in the in vitro derivation of primordial germ cells and gametes, as well as the promise of gonadal stem cells. Embryo models and organoid systems provide a further opportunity to explore germ cell specification and maturation within a proxy ‘in vivo’ context. Additionally, stem cells enable high-throughput molecular studies to explore the mechanisms underlying fertility-related diseases. Using the international effort to derive gametes from stem cells of the Northern White Rhino as a case study, we highlight the contribution of alternative mammalian systems within the field of assisted reproductive treatments and point toward promising directions for future research. Contextualizing stem cell derivation and differentiation within a comparative species framework will be essential for harnessing the full potential of reproductive technologies for human fertility while simultaneously opening new frontiers in wildlife conservation.

Facing the decline in human fertility and an increased need for assisted reproductive technologies (ARTs), the opportunity to leverage insights from diverse species is rarely taken (1, 2). Despite the vast array of reproductive mechanisms across the animal kingdom, reproductive biologists limit their source of knowledge to a handful of model species that may not always correspond well to human reproductive processes (1). The rapidly evolving field of stem cell technology provides an unprecedented opportunity to model gamete development and other relevant reproductive phenomena from a wide range of organisms (3).

Understanding developmental mechanisms, especially across species from the amniote phylum, may reveal species-specific nuances in signaling pathways potentially critical for human reproduction. The current extinction crisis is promoting interest in these technologies to preserve the reproductive capacity of endangered species, offering valuable insights into gametogenesis and fertility beyond the standard mouse model (4). These efforts could not only advance conservation but also enable comparative biological approaches to enhance technical optimization and support emerging possibilities within human ART (Fig. 1).

**Figure 1:**
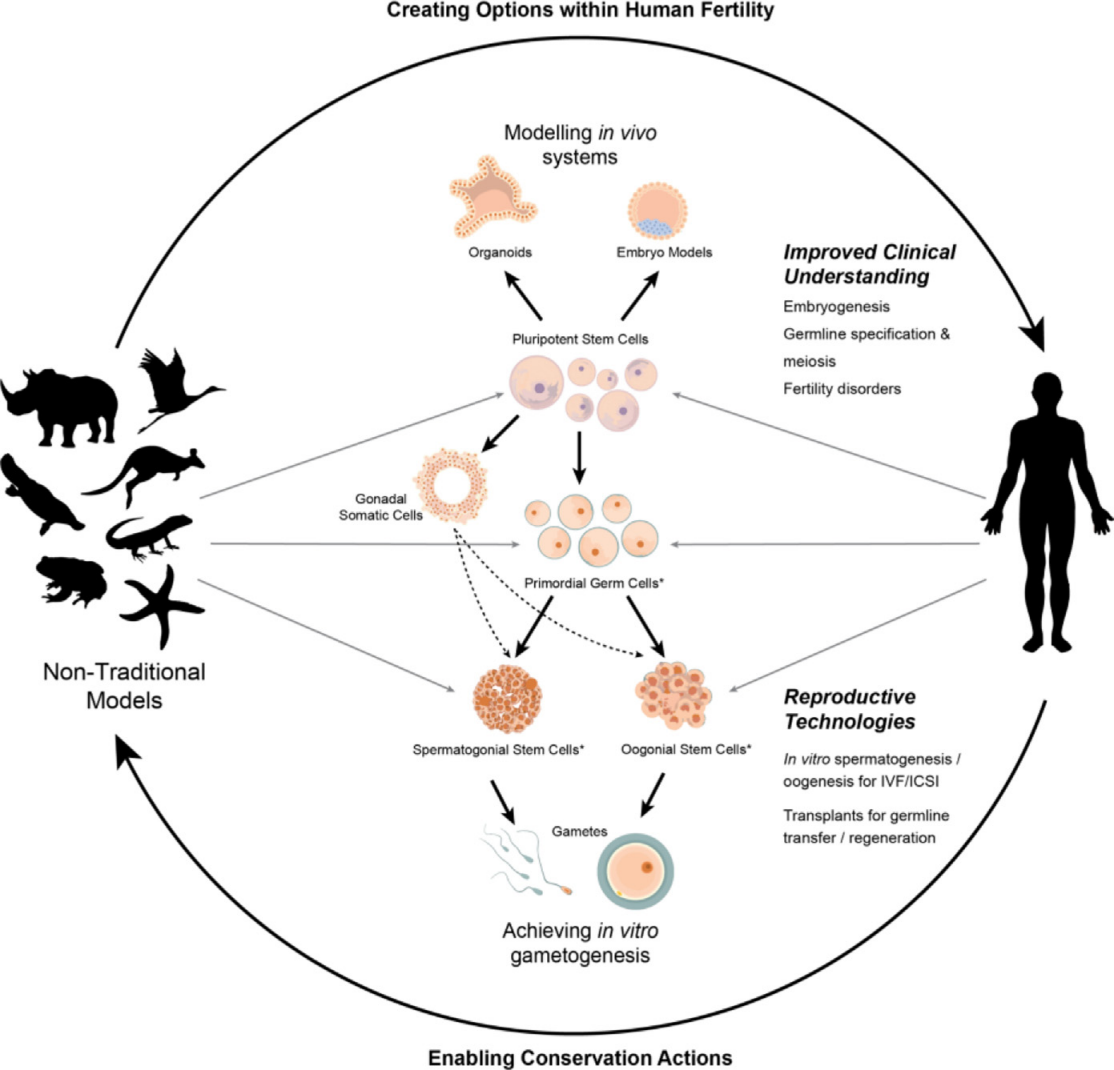
Harnessing comparative biology to elucidate stem cell mechanisms could enable advances in human fertility and conservation. Induced pluripotent stem cells (iPSCs) can be derived from somatic cell lines and converted to any cell type, including gametes, supporting somatic cells, or gamete-precursor stem cells. iPSCs also offer a way to study in vivo systems via 3D stem cell-derived models, delivering potential insight into fertility disorders or diverse reproductive strategies. Germline stem cells can also be isolated directly from an individual as primary cultures, creating further opportunities to support ARTs. Insights from human stem cell technology can be leveraged to advance conservation efforts; however, human fertility research could also benefit from examining stem cell mechanisms in diverse species. A comparative approach facilitates far greater analytic power and will be mutually beneficial within these respective fields.

The ability to generate gametes from induced pluripotent stem cells (iPSCs) in mice has opened transformative possibilities for human fertility treatments, suggesting functional spermatozoa and oocytes could potentially be produced from a patient’s own somatic cells (5). However, this achievement is still unreplicated in humans, although a lot of progress has been made (6). The regulatory mechanisms driving gametogenesis, including cross-talk from somatic cells within the niche, remain to be elucidated (7). Stem cell-based comparative models could provide essential insights into these processes.

In fact, stem cell technology is emerging as a tool to explore a range of reproduction-related challenges. The presence of a spermatogonial stem cell pool within the testis throughout adult life provides an opportunity to restore sperm production for patients undergoing cancer treatment that depletes germ cells, as well as a potential route to produce sperm in vitro from isolated spermatogonia (8). Again, despite successful experiments in other models, clinical translation is delayed (9). Meanwhile, in the human ovary, the germline progenitor pool is widely accepted as exhausted by birth (10); however, this is not always the case in other species (11). Investigating oogonial regeneration and persistence could reveal pathways to potentially encourage this response in our own species.

Expanding the scope of animal models for reproductive research offers an untapped opportunity to advance ART and fertility treatments. By incorporating diverse model systems, conserved and species-specific regulatory mechanisms essential for germline specification, meiotic progression, and early embryo development can be identified. Additionally, modeling species-specific variations in the reproductive tract could shed light on key phenomena such as diapause and sperm storage. Importantly, genetically varied wild species may provide more informative and robust results than inbred laboratory or agricultural model species. Here, we propose that deploying a comparative approach incorporating diverse species could enable advancements in human reproductive technologies, as well as provide essential options for wild species under threat.

## Human in vitro gametogenesis

### Where can nontraditional models bridge the gap?

Advances in generating human gametes using pluripotent stem cells (PSCs) through in vitro gametogenesis (IVG) are making significant strides (5). Partial reconstitution of human gamete production for both sexes opens exciting possibilities for applications in human developmental biology research, ARTs, and reproductive freedom. However, considerable technical hurdles remain for the full achievement of IVG to produce fertilizable gametes in humans (6). Although demonstrating crucial proof-of-concept, the murine model may not be optimal for informing human IVG because of developmental differences between these species (12). Other conventional models in this area typically include nonhuman primates, and agricultural species (13); however, full reconstitution of IVG remains elusive. Integrating data from a diverse array of nontraditional models could provide clearer insights into the mammalian signaling requirements for gametogenesis, specifically regarding the timing, concentration of inducers, and culture conditions.

One significant roadblock to IVG in primates is inducing meiosis in vitro. In mice, coculture of primordial germ cell-like cells (PGCLCs) with ovarian somatic cells either from fetal mouse ovaries or derived from iPSCs has been required to induce meiosis (14). Despite the importance of supporting somatic cells in regulating gametogenesis, there is an acknowledged gap in the literature for mammalian models other than mice (7). Taking other amniote clades into account, the observed variation in the origin of gonadal cells for avian species suggests that there may be distinct differences between taxonomic groups (7). Defining regulatory differences could enhance the generation of human-supporting cells through the identification of transcription factors that stimulate differentiation to these cell types. Currently, granulosa-like cells have been derived from human iPSCs by overexpressing NR5A1 and RUNX1/2, but coculture proved insufficient to induce meiosis in iPSC-derived PGCLCs (15). A systematic and comprehensive characterization of somatic cells in the mammalian niche could identify novel mechanisms or unknown factors that improve meiosis induction in vitro (7).

In female mice, retinoic acid (RA) provided by fetal ovarian cells activates the meiotic process, whereas in males, RA also drives the differentiation of spermatogonia (16). For primates, the most successful report of stem cell-derived germ cells in vitro has been achieved in cynomolgus monkeys by culturing PGCLCs with mouse ovarian somatic cells to induce zygotene stages with corresponding epigenetic maturation (17). However, this is unreported in humans, suggesting that species-specific differences prevent direct translation of protocols. The role of RA signaling in modulating the onset of meiosis is likely conserved for amniotes, supporting reports that turtle ovarian stem cell-like lines express meiotic markers in vitro in response to RA (18). A cross-species comparison will be an important consideration for understanding which components of this pathway may be essential for human IVG. Here, conserved factors are likely to underlie canonical processes, whereas differences can be linked to evolutionary divergence, providing a phylogenetic and reproductive context for human meiosis. Examining the timing of meiotic entry across species could be used to map out temporal cues essential for germ cell maturation, which could then predict human-specific timing for RA delivery. A similar comparative approach has been used to elucidate recombination during meiosis, with the tiger emerging as a candidate model for studying crossover interference (19). Similarly, capitalizing on the evolutionary split between eutherian and marsupial lineages has yielded valuable information about meiotic progression (20).

### Leveraging multispecies 3D stem cell models

To recapitulate gametogenesis in vitro*,* it will be necessary to reproduce complex in vivo signaling in both sequence and duration (12). Human IVG optimization can require long-term inducer treatments, questioning whether slow germ cell progression mirrors natural gonadal development. 3D stem cell-derived models from species with varying developmental tempos deliver a way to define the temporal signaling that initiates both germline specification and meiosis (21), as well as compare differentiation efficiency. By generating embryo and organoid models, PSCs enable the examination of varying embryogenesis rates and regulation across species where gametes and embryos are difficult to access. Here, taking a comparative approach has shown value: when exploring developmental kinetics, iPSCs from 6 species, including the southern white rhinoceros and human, were used to model the segmentation clock, producing scaling laws informed by embryogenesis duration across species (21). Comparing more species established stronger correlations than would have been possible if only mouse and human models were used. Similarly, by including porcine and primate species, models of epiblast development uncovered conserved regulation of Primordial germ cells (PGC) specification in diverged mammalian species, revealing robust early fate specification processes usually unavailable for study (22).

Additionally, loss- and gain-of-function experiments investigating gene regulatory networks across the amniote phylum driving PGC specification are made possible using stem cell models, reducing the need for whole animal systems. Coculture experiments and other multiorganoid systems could inform research into implantation pathways across species, providing insight into recurrent miscarriage. Such systems also offer a route to explore the phenomenon of diapause that occurs for some eutherian species, such as deer, as well as marsupials and squamates (23), with implications for long-term embryo storage.

Currently, research addressing human fertility disorders relies on a narrow set of model organisms, such as mouse, primate, chick, zebrafish, and *Xenopus*. This limitation may hinder advances in understanding numerous disorders. Nonmodel organisms already reveal mechanisms underlying congenital malformations in humans (24). This potential could be vital for advancing human ARTs. Viviparity, in fact, has evolved more than 100× in squamates alone (25). Investigating placentation evolution and fetal membrane development across the amniote phylum could deepen understanding of human-specific and universal mechanisms. Because many species are not readily available and often endangered, iPSC-derived trophoblast organoids could support high-throughput studies for comparative placentation analyses. Modeling the female reproductive tract might enable research into placentation disorders, as well as sperm storage. Observed for some marsupials (26), this phenomenon has also been reported in reptiles, ranging from seasonal sperm storage in anole lizards to multiyear storage in rattlesnakes (27). Mechanisms behind prolonged sperm storage, along with sperm activation, could be studied by using stem cells to model in vivo conditions, potentially benefiting ARTs.

### Germline stem cells

#### Primordial germ cells

As multipotent stem cells, PGCs are specialized progenitor cells established early in embryonic development, developing into later stages of germline stem cells. Accumulating evidence indicates that the mechanisms of early human development and cell fate specification are conserved for species with bilaminar disc embryos rather than the egg cylinder embryos produced for mice (22). However, the molecular mechanisms of germ cell development in nonmodel mammalian species remain largely unknown, reinforcing the importance of studying human germline development in the context of other mammals (12, 28). Given the similarities in regulatory mechanisms between bilaminar disc species, in vitro methods building on human research have been useful for achieving generation of PGCLCs for other bilaminar disc species, including cynomolgus monkey, rabbit, pig, and white rhinoceros (29) (BOX 1). As murine and human PGCs develop differently (12), a global understanding of PGC specification across clades is essential to identify universal factors and species-specific distinctions, such as in WNT and BMP pathways. Improved stem cell understanding may require support from computational modeling (30), as currently, a limited number of species are readily accessible for study.

**Box 1:** 


CASE STUDY: Deriving Primordial Germ Cells for the Northern White Rhino
The white rhinoceros (*Ceratotherium simum*) includes 2 subspecies, the southern white rhinoceros (SWR; *C. simum simum*) and the endangered northern white rhinoceros (NWR; *C. simum cottoni*), with only 2 females left on Earth. Although recovery of the NWR will depend on the application of stem cells and ARTs (31), little is known about rhinoceros gametogenesis. The San Diego Zoo Wildlife Alliance has a unique resource, the Frozen Zoo, which contains viable cryopreserved fibroblasts from >11,000 individuals, including 12 individuals of NWRs. The 12 NWR cell lines retain higher genetic diversity than individuals from the current SWR population, and the genomes of NWR cell lines can mitigate inbreeding depression from genetic load (32), delivering hope for the restoration of a healthy rhino population.
Those cryopreserved fibroblasts were reprogrammed into iPSCs with primed-type characteristics (33). Northern white rhinoceros-iPSCs, as well as SWR-embryonic stem cells (ESCs), possess the capability to differentiate into PGCLCs that express PGC-associated transcripts, including *BLIMP1*, *TFAP2C*, *SOX17*, and *NANOS3*. Interestingly, rhino PGCLCs are transcriptionally more similar to humans than are PGCLCs from mice (29). SOX17 was found to be essential to induce germline specification from PSCs for the rhino, human, cynomolgus monkey, and others; however, this has not been observed for rodent models (22). It is, therefore, likely the role of SOX17 in PGC specification represents the ancestral condition, and that this pathway has diverged in the rodent lineage.
The rhino provides an unexpectedly valuable resource for human fertility research. Not only are there similarities between germ cell regulation, but as a large animal model, this species provides other parallels, such as slower gestation time. Furthermore, the availability of ESC lines makes for robustly validated iPSCs, strengthening the reliability and translatability of differentiation data. There are few species for whom this condition can be met.
Importantly, generation of rhino PGCLCs was achieved without in vivo rhino PGC data, which is used as a reference in standard mouse and human studies. In such cases, where in vivo reference knowledge is absent, comparative studies can be a powerful tool. Here, similarities in germ cell development between rhinos and humans supported the successful induction of PGCLCs for this highly endangered species. In an upcoming study, iPSC lines from 7 NWRs and 3 SWRs, as well as 2 human lines, were differentiated into PGCLCs using two different protocols (Unpublished). This study will further elucidate differences between species, sex, and individual donors, as well as mechanisms of PGC development in both rhinos and humans.

#### Spermatogonial stem cells

As part of the goal of producing gametes, PGCs can be differentiated into intermediate stem cells along male or female pathways. In the male germline, spermatogonial stem cells (SSCs) offer a more direct route for fertility preservation and restoration, because they are already committed to becoming spermatozoa. When transplanted into the murine testis, SSCs can repopulate the seminiferous tubules providing hope for prepubertal patients undergoing chemotherapy, which often results in infertility (8). Testicular tissue grafting offers another approach for restoring spermatogenesis in these patients (34), whereas SSCs could also be differentiated in vitro to produce sperm, presenting new fertility treatment avenues (35).

Despite routine murine SSC culture, significant gaps remain in our understanding of their molecular regulation. Although long-term culture is established for mouse, human cultures require optimization (36). This limits SSC expansion from testicular biopsies and makes it difficult to correlate human and murine regeneration after testicular injury. In vivo, spermatogonial regulation depends on signaling from supporting somatic cells in the niche (37). Notably, differentiation-destined progenitor cells can re-enter the self-renewing pool in response to signaling changes (38). As such, the testis regulatory environment is an ideal model for studying stem cell manipulation and the balance between self-renewal and differentiation. However, distinct culture requirements for murine SSCs across strains suggest that genetically diverse animal models may yield improved insights into conserved SSC regulation (14). Studies in tree shrews and peccaries have explored how signaling pathways and supporting cell distribution impact SSC expansion and regulation (39, 40). These insights, along with findings from more distant species like the scorpion mud turtle, suggest conserved markers for self-renewal in the testis (41). Examining the SSC transcriptome across 7 mammalian species has revealed transcriptional heterogeneity in spermatogonia, which varies across both species and developmental stages (42). Here, cross-species comparisons were used to explore how developmental and reproductive traits drive the evolution of germ cell regulation. Additionally, single-nucleus transcriptomics for spermatogenic cells in primate testes, alongside mouse, marsupial, monotreme, and an avian outgroup, have been used to establish a comparative resource for studying mammalian fertility disorders (43). Such cross-species studies could elucidate factors for human SSC expansion, including growth factors, hormones, cytokines, and metabolic requirements.

Although murine spermatogonial populations may differ from those of primates (44), felids, such as domestic cats, cheetahs, and Amur leopards, show conservation of mammalian SSC markers (PLZF, POU5F1, and UCHL1) and are recommended for studying sperm abnormalities and oocyte-related fertility preservation (1, 45). Meanwhile, the endangered Indian black buck displays NANOG and SSEA1 in spermatogonia, with POU5F1 restricted to more differentiated cells (46). Humans have a reserve quiescent population that is not observed for rodents, however is reported for pigs, representing important models for human disease (47). Comparative studies of human, mouse, and opossum spermatogonia reveal the expression of shared genes, indicating some fundamental conservation of genetic regulation for SSCs between eutherian and marsupial lineages (8). Further comparative studies will improve the understanding of conditions that promote mammalian spermatogonial maintenance in vitro.

For endangered species, biobanking of spermatogonia or testicular tissue preserves male genetic material for potential reintroduction. Although sperm cryopreservation requires complex species-specific optimization, spermatogonia can be frozen using standard protocols (45). Extracted SSCs could be differentiated into spermatozoa in vitro or auto-/xeno-transplanted to a more prolific host. For example, SSCs of the endangered ocelot will proliferate in the domestic cat testis, providing a way to increase sperm availability for an individual (48). Xenografting testis tissue into sterile immunodeficient mice has induced varying degrees of spermatogenesis for endangered species, including banteng, bison, white-tailed deer, Mohor’s and Cuvier’s gazelles, and Iberian lynx (34). Autologous transplants offer a means to restore spermatogenesis for prepubertal chemotherapy patients, although success is so far limited to nonhuman primates (9, 34). Human xenografts did not progress beyond the pachytene stage (49), suggesting unknown requirements for spermatogonia. Comparative research provides an opportunity to relate species-specific molecular regulation to the signaling environment to support both in vitro and in vivo maintenance, expansion, and differentiation of SSCs for clinical applications.

#### Oogonial stem cells

Although it has been widely proposed that humans are born with a finite pool of oocytes, there is ongoing debate regarding the continued presence of oogonial stem cells within the ovaries (10). These putative stem cells may be activated under specific culture conditions, but significant research is still required to translate this knowledge into meaningful applications for human fertility (10). In mouse neonatal ovaries, a rare population of stem cells has been identified that can differentiate into oocytes when transplanted into sterile ovaries (50). Notably, mitotically active cells expressing germline markers have been isolated from the ovaries of several mammalian species, although their physiological role remains unclear (51). The ability to generate oocytes for women experiencing premature ovarian insufficiency, or premenopausal patients undergoing chemotherapy, would represent a groundbreaking advancement in clinical fertility practice. To explore this potential, large animal models such as pigs and cattle are being used to isolate cells expressing stem cell markers from the ovary (52). In fact, the southern white rhinoceros reportedly maintains mitotically active germ cells exhibiting stem cell markers in the ovary after birth, highlighting divergent regulation of oogonial persistence in mammalian species (11). However, lessons from more distantly related amniotes could be used to identify candidate pathways for the persistence of the oogonial pool after birth.

One of the most intriguing yet understudied phenomena in herpetology is the regenerative ability of many reptiles to regrow their entire reproductive tracts after spaying, provided that a small portion of ovarian or uterine tissue remains (53). This liver-like regeneration could benefit human ARTs, potentially restoring fertility for cancer survivors. iPSC-derived ovarian organoids may offer a way to explore the molecular basis of these regenerative properties. Ovarian stem cell-like lines from 2 species of juvenile turtles have been isolated and cultured (18, 54), potentially providing the basis for in vitro models for other reptiles. In evolutionarily distant vertebrates such as amphibians and fishes, some species appear capable of female germline regeneration (55, 56) or even sex switching although the mechanism behind this transition remains unclear (57). Research into stem cell regulation in echinoderms and other marine deuterostomes aims to model regenerative processes for clinical translation (58). Understanding how ovarian stem cell niches regulate self-renewal across species and how reproductive aging varies in certain mammals (11) will be important for reproducing these types of pathways.

## Discussion

Reconstituting the full germline cycle in vitro for humans and other species is anticipated to be one of the most transformative advances for biological research. With the ability to convert somatic cells to pluripotency has come an array of new possibilities in human health. Using these cells to produce gametes could overcome many of the challenges currently faced by patients with fertility issues while also opening new possibilities previously out of reach. Although the achievement of murine gametes from iPSCs indicates this outcome is possible, incorporating lessons from other species may be necessary to surmount roadblocks.

Although case studies remain limited, the Northern White Rhino strategy exemplifies a dedicated, cross-institutional effort to reconstitute the entire germline cycle for a nonmodel species. Such advances, driven by urgent conservation needs, should be leveraged to inform human fertility research. Yet, the scarcity of examples for wild species is concerning, given their potential. In addition to mitigating the extinction crisis, in vitro gametogenesis could reveal key aspects of germline specification, pluripotency, and developmental evolution. Organoids and embryo models further offer valuable platforms to explore diverse biological systems with implications for human health.

Comparative studies of SSCs across species highlight the potential of spermatogonial and testicular transplantation techniques, providing hope for fertility preservation. Concurrently, the phenomena of ovarian regeneration and oogonial persistence observed in some species suggest opportunities to understand and potentially extend the lifespan of the oogonial pool in humans. Modeling the reproductive tract provides further possibilities for leveraging advantageous reproductive mechanisms.

Although much of this technology is nascent, traditional models provide fundamental information as a basis for developing working methods in less understood species. However, this benefit necessarily flows both ways, and the value of examining diverse developmental and reproductive mechanisms cannot be understated (Fig. 1). Emerging computational tools offer promising ways to decipher stem cell biology (30) and predict the molecular pathways underlying infertility, especially for hard-to-access species. Factoring in molecular differences at diverged loci could be used to explain barriers in applied stem cell technologies, benefiting clinical translation and transforming reproductive options for endangered wildlife. With reproductive rates declining across many species, an integrative approach to fertility research promises mutual gains for conservation and reproductive health.

## Declaration of Interests

A.M.H is a Program Manager at Revive & Restore, committed to engaging the scientific community on the value of including stem cell technology within wildlife conservation. A.W. has nothing to disclose. Y.E has completed a postdoctoral position developing stem cell technologies for the NWR with the SDZWA. R.B. is an Associate Director at Conception Bio.
